# The use of Hardy–Weinberg Equilibrium in clonal plant systems

**DOI:** 10.1002/ece3.1946

**Published:** 2016-01-25

**Authors:** Vladimir Douhovnikoff, Matthew Leventhal

**Affiliations:** ^1^ Biology DepartmentBowdoin College6500 College StationBrunswickMaine04011

**Keywords:** Clonal plant, Hardy–Weinberg Equilibrium, heterozygosity, PD value

## Abstract

Traditionally population genetics precludes the use of the same genetic individual more than once in Hardy–Weinberg (HW) based calculations due to the model's explicit assumptions. However, when applied to clonal plant populations this can be difficult to do, and in some circumstances, it may be ecologically informative to use the ramet as the data unit. In fact, ecologists have varied the definition of the individual from a strict adherence to a single data point per genotype to a more inclusive approach of one data point per ramet. With the advent of molecular tools, the list of facultatively clonal plants and the recognition of their ecological relevance grows. There is an important risk of misinterpretation when HW calculations are applied to a clonal plant not recognized as clonal, as well as when the definition of the individual for those calculations is not clearly stated in a known clonal species. Focusing on heterozygosity values, we investigate cases that demonstrate the extreme range of potential modeling outcomes and describe the different contexts where a particular definition could better meet ecological modeling goals. We emphasize that the HW model can be ecologically relevant when applied to clonal plants, but caution is necessary in how it is used, reported, and interpreted. We propose that in known clonal plants, both genotype (GHet) and ramet (RHet) based calculations are reported to define the full range of potential values and better facilitate cross‐study comparisons.

## Introduction

One of the most commonly used mathematical models in population genetics is the Hardy–Weinberg Equilibrium (HWE). It is used to predict genotype and allele frequencies in future generations, assess the equilibrium of current populations, and interpret the genotype and allele frequency of earlier generations. All understood as having important ecological and evolutionary implications.

Facultative clonal plants are inherently problematic subjects for the application of the model. Depending on the degree of clonality, there are several assumptions the model calls for that they often do not meet including:

1. *Sexual reproduction*. To varying degrees, sexual reproduction may be involved in population maintenance and growth.
2. *Nonoverlapping generations*. Life spans of clonal plants are as extreme as possible. This makes the concept of generations problematic. Some genotypes will live hundreds if not thousands of years, while others may be short‐lived.
3. *Large populations*. In the most extreme cases, clonality can result in a genotype‐based population of 1. When a habitat is finite, added clonality will tend to reduce population size.
4. *Equal allele frequencies in the sexes*. In dioecious plants, clonality can result in an uneven male/female representation.
5. *Diploidy*. Polyploidy is common in clonal plants.

Despite the failure to meet these assumptions, the model is still found to be informative, and values such as expected heterozygosity or fixation index in clonal plant systems are commonly reported. In other circumstances, the model might be applied to a plant species where the extent of clonality is not recognized. In both situations, it is important to acknowledge the potential variation in results that could stem from varying levels of clonality and the way the HW model is applied.

Some of the most thorough work modeling the population genetics of clonal organisms are founded in nonplant systems (Meeus et al. 2006, Helkett et al. 2005, Prugnolle et al. 2005, Balloux et al. 2003). These models make important progress in interpreting and predicting clonal population dynamics considering factors such as intermittent sexual reproduction, migration, sex, life cycle, inbreeding, and coancestry. More commonly, clonal studies are limited in modeling scope and rely primarily on simple HW‐derived statistics based on estimates of allele frequency. The goal of this study was to better understand the range of potential outcomes using this more limited and simplified framework.

The fact that each genotype (clone) can be made up of multiple genetically identical but potentially independent units (ramets) poses considerable problems. HW values can be derived based on estimates of allele or genotype frequency. Inherent in this estimate is the necessity to define the sample unit, commonly assumed to be one sample datum from each individual. However, in clonal plants the definition of the individual is not a simple matter. Is the individual each potentially independent biological unit and thus each ramet within a clone? Or is the individual represented by the single genetic profile shared by all genetically identical ramets (genet)? Not surprisingly, as we will discuss here, the definition used can have important implications for estimates of heterozygosity.

In nonclonal applications of HW, there is an expectation that each individual is a genetically distinct entity (genotype). In clonal plant systems, ecologists have not strictly adhered to a one sample per genotype approach for various reasons. For example, assuming all other HW assumptions are met, a departure from equilibrium using a ramet‐based definition has been used as a measure of clonality (Halkett et al. 2005, Stenberg et al. 2003). Also, an argument can be made that restricting calculations to a single ramet per genotype confounds the HW random mating assumption. When clonal size distributions are skewed, such as when a single clone dominates a population, counting an entire clone as a single datum severely discounts it modeled contribution to the next generation.

In a cursory survey of clonal plant studies with HW‐derived statistics (Table 1), we observed studies that include all sampled stems regardless of genetic identity (Young et al. 2002; Lexer et al. 2005; Suvanto and Latva‐Karjanmaa 2005; Travis and Hester 2005; Stamati et al. 2007; Lambertini et al. 2008; Honnay et al. 2010; Tanaka et al. 2011; Lauron‐Moreau et al. 2013; Sochor et al. 2013; Perdereau et al. 2014), some that attempt to limit genet replication by establishing a minimum sampling distance (Pluess and Stöcklin 2004; Alsos et al. 2009; Jiménez‐Mejías et al. 2012), studies that only include one stem per known genotype (Nagamitsu et al. 2004; Lhuillier et al. 2006; Beatty et al. 2008; Schonswetter et al. 2008; Pollux et al. 2009; Rathmacher et al. 2009; Meloni et al. 2013; Berlin et al. 2014; Chung et al. 2014), studies that report results for both approaches (Stenstrom et al. 2001; Vaughan et al. 2007; Lin et al. 2009), and others that are not explicit in their approach (Jones and Gliddon 1999; Smulders et al. 2008; Steltzer et al. 2008; Trybush et al. 2012). In this study, we explore and contrast the dynamics of HW using the ramet‐ and genet‐based definitions. We investigate cases that demonstrate the extreme range of potential outcome differences. We describe below the different contexts where each definition could better meet modeling goals. We emphasize that the HW model can be ecologically relevant when applied to clonal plants, but caution is necessary in how it is used, reported, and interpreted.

**Table 1:** Cursory survey of clonal plant studies with HW‐derived statistics

Sampled all stems	One sample per genotype	Ramet and genotype approach reported	Estimated minimum distance	Not explicitely described
Perdereau et al. (2014), Lauron‐Moreau et al. (2013), Sochor et al. (2013), Tanaka et al. (2011), Honnay et al. (2010), Lambertini et al. (2008), Stamati et al. (2007), Suvanto and Latva‐Karjanmaa (2005), Travis and Hester (2005), Lexer et al. (2005), Young et al. (2002)	Chung et al. (2014), Berlin et al. (2014), Meloni et al. (2013), Pollux et al. (2009), Beatty et al. (2008), Rathmacher et al. (2009), Schonswetter et al. (2008), Lhuillier et al. (2006), Nagamitsu et al. (2004)	Lin et al. (2009), Vaughan et al. (2007), Stenstrom et al. (2001)	Jiménez‐Mejías et al. (2012), Alsos et al. (2009), Pluess and Stöcklin (2004)	Trybush et al. (2012), Smulders et al. (2008), Steltzer et al. (2008), Jones and Gliddon (1999)

## Methods

While many parameters are derived from the HWE model, for the purposes of this discussion we will focus on measures of heterozygosity, one of the most commonly reported and broadly recognized. To observe the influence of clone size on calculations of heterozygosity, we incrementally expanded the representation of a single clonal genotype in a hypothetical population of one thousand stems. Initially, all stems were genetically distinct individuals starting at HWE. This is not a model of population development, but a representation of a range of clonal structures and HW‐derived heterozygosity values. For a single locus, both expanding homozygous clonal genotypes and expanding heterozygous clonal genotypes were derived. A constant sample size (1000) was maintained with nonclonal individuals replaced by clonal ramets as a proportion of the total population (all genotypes have an equal likelihood of being dropped from the population except the expanding clone). In this simplified system, only a single genotype was clonally expanded in each trial. The dropped individuals also include those with the same genotype as the expanded clone genotype. Scenarios were run until at least one ramet remained for each genotype.

**Figure 1:**
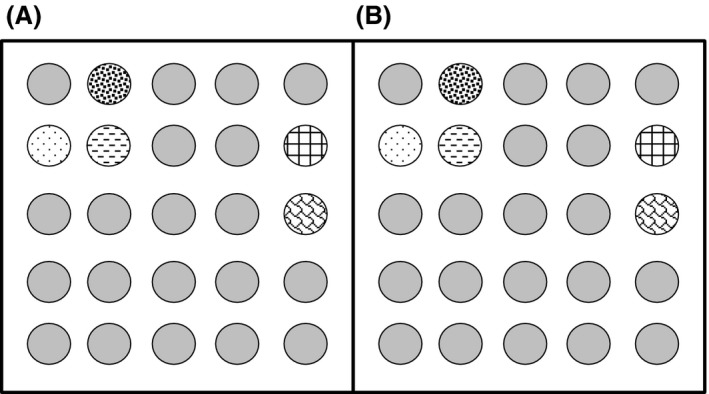
(A) A population of 25 stems counted as ramets. The sample size would be *N* = 25. Each circle is a ramet. (B) A population of 25 stems counted as genets. The sample size would be *N* = 6. Each texture represents a different genet.

Expected heterozygosity of ramets is referred to as RHet. Allele frequencies were estimated (Table 2) including all ramets regardless of genetic identity (Fig. 1A). Expected heterozygosity of ramets was measured using the HW calculation 2pq, with p and q being the alternate allele frequencies, respectively. The procedure was repeated for populations of the same size with a range of starting values for p (p = 0.5, p = 0.4, p = 0.3, p = 0.2, p = 0.1, p = 0.01).

**Table 2:** Homozygous clone of various sizes. With each incremental increase in clonality (PD value), heterozygosity values were calculated. For genotype counts, sample size of 1000 was maintained by incrementally replacing nonclonal genotypes with each added clonal ramet. Genotype counts based on the genet model (pp‐genet) only counted a given genotype once, and larger clones resulted in a reduced count

Genotypes (1000 sampled)				Ramet model (frequencies)						Genet model (frequencies)			PD value
qq	qp	pp	pp‐genets	p	p2	q	q2	Exp. Het.	Obs. Het.	p	q	Exp. Het.	
250.0	500.0	250.0	250.0	0.50	0.25	0.50	0.25	0.50	0.50	0.50	0.50	0.50	1.000
202.5	405.0	392.5	202.5	0.60	0.35	0.41	0.16	0.48	0.41	0.50	0.50	0.50	0.810
147.6	295.2	557.1	147.6	0.70	0.50	0.30	0.09	0.42	0.30	0.50	0.50	0.50	0.590
96.9	193.7	709.4	96.9	0.81	0.65	0.19	0.04	0.31	0.19	0.50	0.50	0.50	0.387
51.5	102.9	845.6	51.5	0.90	0.80	0.10	0.01	0.18	0.10	0.50	0.50	0.50	0.206
9.5	19.1	971.4	9.5	0.98	0.96	0.02	0.00	0.04	0.02	0.50	0.50	0.50	0.038

For expected heterozygosity of genets, the sampling method was the same, but each genotype was only counted once (Fig. 1B). The procedure was repeated for the same scenarios as for the ramet method. Expected heterozygosity of genotypes was referred to as GHet.

The commonly used metric for clonality, PD values (percent distinguishable), were calculated for each model as the number of genetically distinct individuals divided by the total number of samples (Ellstrand and Roose, 1987).

In summary, we calculated heterozygosity based on allele frequencies adjusted for changes in clone size following two approaches: (1) Values from all ramets were included in calculations; and (2) one value from each genotype was used in calculations.

## Results and Discussion

In calculations of HW, the relative pool of alleles and genotypes observed will be very sensitive to a ramet or genet definition of the individual. A ramet definition in a clonal population will result in a greater number of individuals being included in heterozygosity estimates, but clonal redundancy will result in greater allele and genotype representation of the largest clones relative to the smaller clones. This can skew heterozygosity calculations depending on the makeup of the clone. The genet method will avoid any clonal redundancy by only including a single ramet among all the clonal replicates, reflecting the standard approach to HW calculations where no genetic individual is included more than once, but it will not account for the ecological footprint of the larger clones.

### Expected heterozygosity

The ramet method and genet method can result in two greatly different estimates of heterozygosity with decreasing PD values (representing greater levels of clonality). Greater clonality will move a population RHet further from GHet. The extent and path this difference follows depends on the size distribution of the genotypes (as % of total ramets) and whether the clonal genotype is heterozygous or homozygous at the locus.

**Figure 2:**
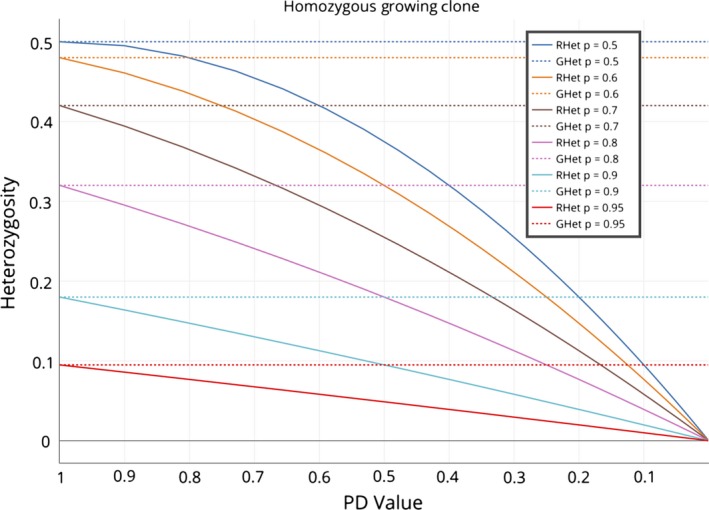
RHet (solid line) and GHet (dashed line) values for stand with incrementally greater proportion of ramets represented by a homozygous clone. Different colors consider scenarios for a range of GHet values. Ghet values are constant as long as there is at least one ramet per genotype, after which values will abruptly correct to zero heterozygosity represented by the homozygous clone.

**Figure 3:**
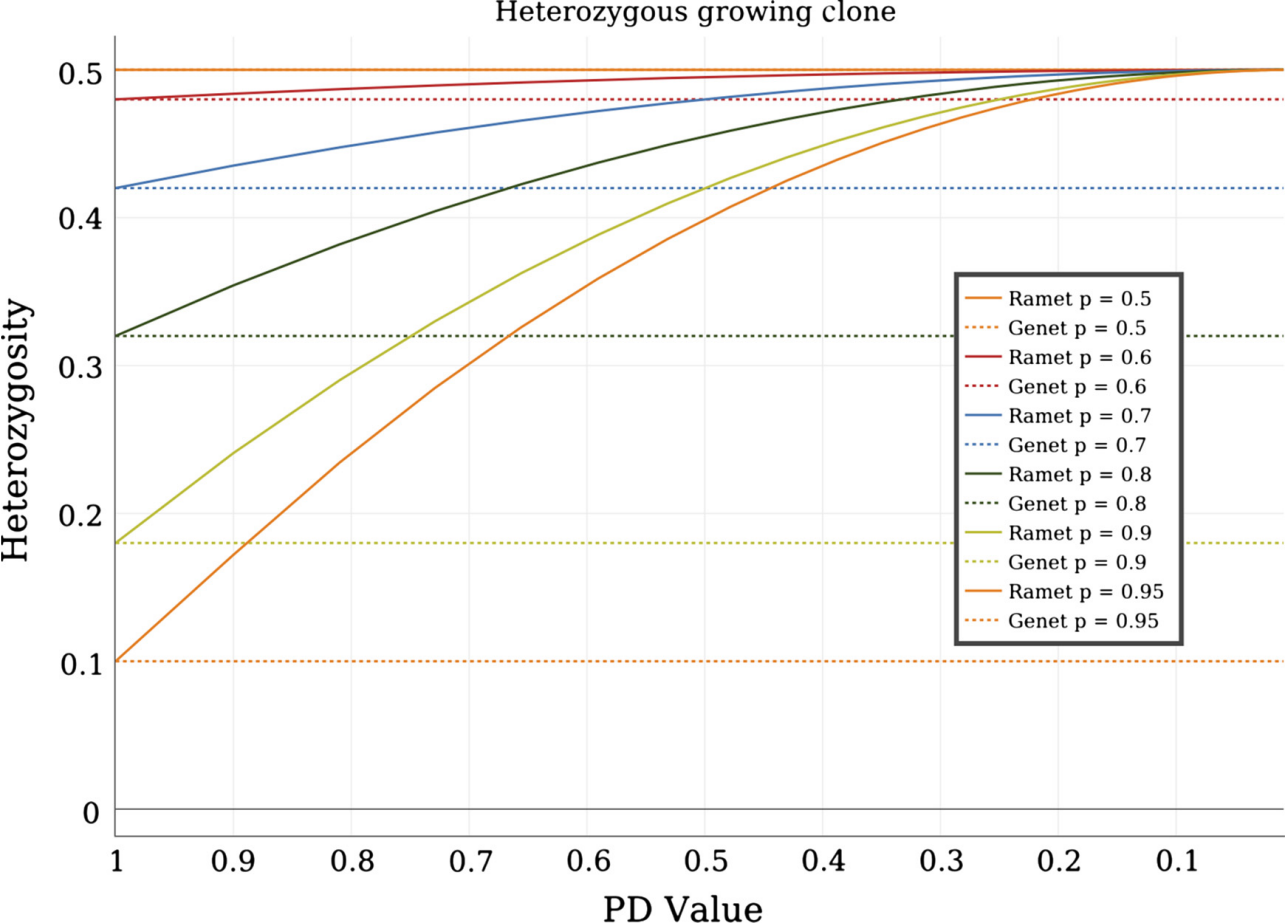
RHet (solid line) and GHet (dashed line) values for stand with incrementally greater proportion of ramets represented by a heterozygous clone. Different colors consider scenarios for a range of initial GHet values. Ghet values are constant as long as there is at least one ramet per genotype, after which values will abruptly correct to 0.5 heterozygosity represented by the heterozygous clone.

With decreasing PD values (larger clones), GHet remains constant because it ignores clonal replicates. This is true until the last representative of alternate genotypes is dropped from the population. When the clonal genotype represents 100% of the remaining alleles, heterozygosity values abruptly swing to 0.5 in the case of a heterozygous clone or 1 in a homozygous clone. In homozygous clones, RHet will gradually decrease at first and accelerate till fixation as clonal representation increases (Fig. 2). In heterozygous clones, RHet will increase rapidly and decelerate till it approaches 100% heterozygosity (Fig. 3).

**Figure 4:**
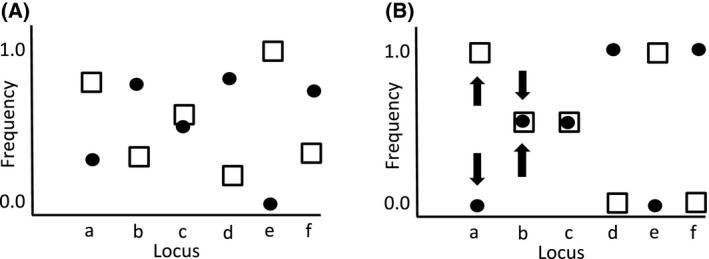
Effect of increased clonality (reduced PD values) on allele diversity (square = p, dot = q). At low clonality levels (A) a range of intermediate allele frequencies are possible. With high clonality (B) homozygote fixation results in reduced allele diversity. Both homozygotes and heterozygotes will trend (↑) towards a single genotype at each locus.

The difference between the GHet and RHet will depend on the size distribution of clones present. On one distribution extreme, as percent distinguishable values decrease, redundant ramets can be evenly distributed among multiple clonal genotypes or on the other extreme fall within a single expanding clone. The greatest difference between RHet and GHet values occurs in the latter case when all homozygous loci approach fixation for one allele, and all heterozygous loci will trend to 0.5 heterozygosity. An even distribution will minimize differences between GHet and RHet with decreasing PD values. In multilocus genotypes represented by both homozygous loci and heterozygous loci, it is important to note that allele diversity will decrease with increased clonality, and the representation of genotypes at each locus will be exaggerated due to both the fixation of homozygotes to one allele and overrepresentation of heterozygotes (Fig. 4).

The degree of influence of PD values on RHet will also vary depending on the related GHet value. RHet distance from GHet will be most exaggerated when a homozygote clone is in a stand of GHet 0.5 (Fig. 2). Each added homozygote ramet will move RHet further from GHet following an exponential curve toward fixation.

For a heterozygote clone, the distance of RHet from GHet is greatest when it is part of a stand with GHet near zero (Fig. 3). With GHet based on almost no heterozygotes and the near fixation of one allele, increased PD values resulting from a heterozygous clone will linearly increase the representation of the heterozygous genotype until homozygotes approach zero. RHet will follow a negative exponential curve from GHet to RHet, approaching RHet of 0.50 when PD values approach zero.

**Figure 5:**
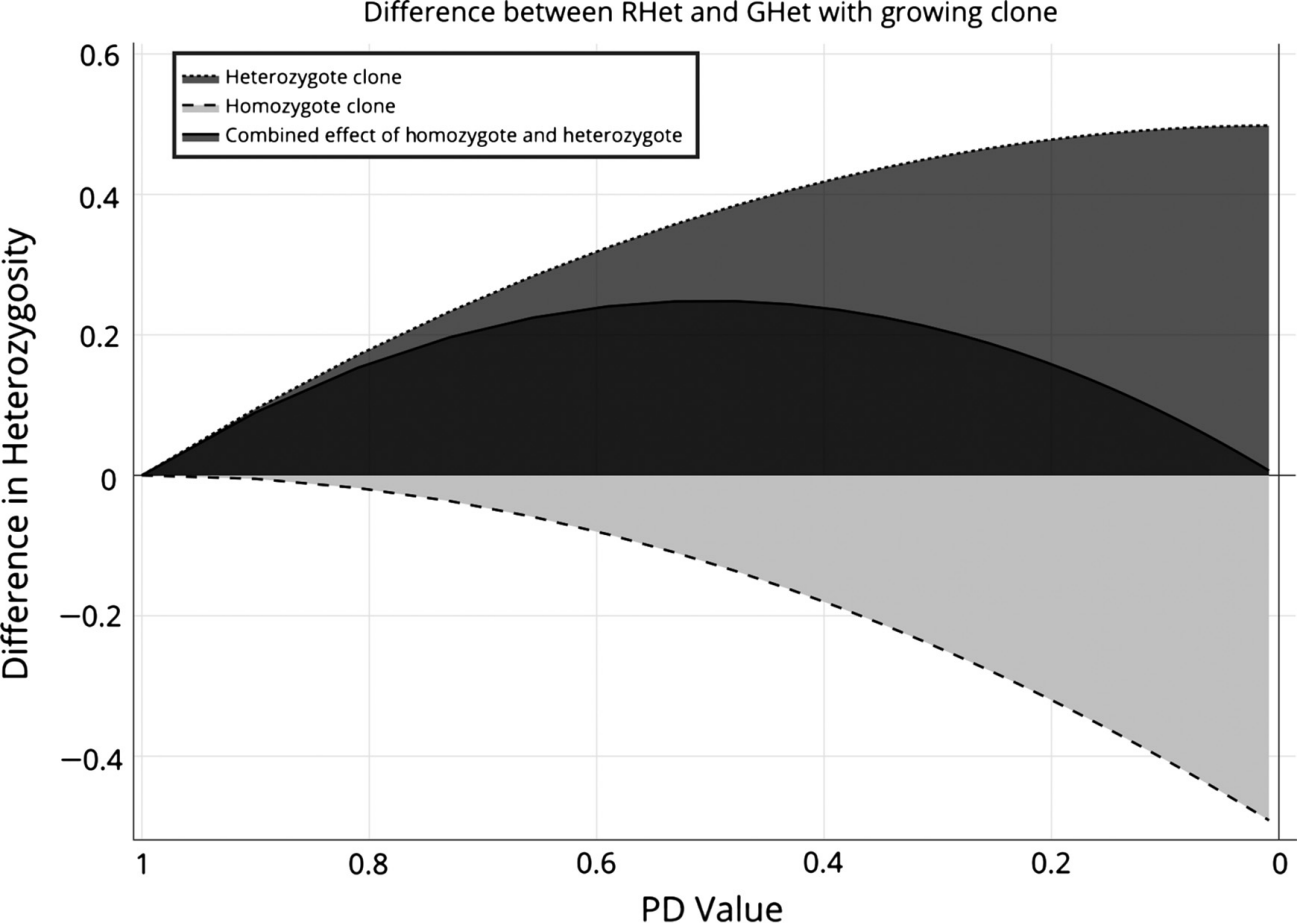
Error in estimated heterozygosity (difference between GHet and RHet), due to inappropriate definition of the individual, at increasing levels of clonality.

The difference between GHet and RHet represents potential range of variation in heterozygosity estimates depending on how HW is applied. It might also be considered the range of error if the definition of the individual used is inappropriate for the question at hand (Fig. 5). As described earlier, the extent of error depends on PD value, size distribution of clonality, the genotype of clones, and the stand GHet. As PD value decreases, heterozygous clones result in decelerating error and homozygous clones result in accelerating error. In both circumstances, the difference between GHet and RHet is the greatest as PD values approach zero. The interaction of these two curves may be relevant in understanding the combined effect when multiple clones, both heterozygous and homozygous for a particular locus, occur, or when considering multilocus genotypes represented by both homozygous loci and heterozygous loci. It appears that if heterozygous and homozygous loci are roughly equal in representation, the combined effect on heterozygosity error would be greatest at intermediate levels of clonality.

### Which model when

With the potential for broad differences in heterozygosity value depending on which model is used, it is important both to carefully select the model applied and explicitly state which was used. A genet model approach will be appropriate in situations where the unit of interest is the representation and influence of the genetic individual assuming equal probability of successful reproduction or in situations where relative size is not relevant such as calculations related to richness or retrospective studies that are interested in the heterozygosity of the stand founders. However, the ramet model may be more appropriate in situations where the structure of the genepool is relevant. Compounded genetic representation of clonal ramets can influence the expected heterozygosity if clonality adds to the probability of allele representation in the next cohort. Many studies that model projected outcomes would benefit from inclusion of a ramet model.

## Conclusions

HW model is powerful and extremely useful, but tricky when used in a clonal system.

Maximum model variation does not necessarily occur at the extreme PD values. Under some conditions, intermediate PD values can result in greater variation. Factors that will influence the extent of variation when different definitions of the individual are used in a clonal system include the following:

1. Clone size distribution (most extreme case tested in this study).
2. The genotype of clones.
3. GHet.
4. The life history of clones that determine the genetic contribution of each ramet (dioecious vs monecious, ramet longevity, distribution of reproductive resources across ramets).
5. Spatial distribution of ramets and clone structure (aggregations can limit the distribution of genetic material to other genotypes).

Traditional population genetics precludes the use of the same genetic individual more than once in Hardy–Weinberg‐based calculations due to the model's explicit assumptions. However, ecologists can find calculations that do so informative in circumstances where the scale of genetic representation is of primary relevance, and it is important to satisfy the random mating assumption. For example, when a replicated and dominant genotype has a disproportionate influence on the genepool and subsequent cohorts, which is not unusual in clonal populations. In cases where clone size distribution is skewed, the use of the genet model might result in analysis suggesting greater diversity than actually exists. There are also inherent interpretive risks in the ramet model such as the inaccurate impression of inbreeding. In any case, when working with clonal plants different definitions of the individual can result in extremely different results and therefore must be addressed *a priori* and to best meet research goals. Furthermore, the research audience should be clear on potential differences when comparing values across studies. For these reasons, we propose that results based on both ramet‐ and genet‐based models be reported representing the band of potentially accurate estimates.

Of the many parameters derived from the HWE model, this discussion focuses on heterozygosity, one of the most commonly reported and broadly recognized. Further work exploring the influence of clonal plant structure on the breadth of HWE‐based parameters is still necessary.

## Conflict of Interest

None declared.
